# Digital health in allergy care: current practices, evidence, and future prospects

**DOI:** 10.3389/falgy.2026.1760856

**Published:** 2026-04-14

**Authors:** Olga Lourenço, Adriano N. Raposo

**Affiliations:** 1 RISE-Health, Department of Medical Sciences, Faculty of Health Sciences, University of Beira Interior, Covilhã, Portugal; 2 IT-UBI, Instituto de Telecomunicações, University of Beira Interior, Covilhã, Portugal

**Keywords:** allergy care, artificial intelligence, clinical decision support, digital health, machine learning, mobile health apps, telemedicine, wearable devices

## Abstract

This mini-review summarizes the current applications of digital tools in allergy care, including telemedicine platforms, mobile health technologies, and AI-based decision support systems. It critically examines available evidence, benefits, and challenges for clinical practice and outlines future directions for integrating digital health into personalized allergy management. Mobile apps and wearable sensors enable real-time tracking of symptoms, medication use, and environmental triggers, providing actionable data that supports proactive disease management. Continuous data streams enhance clinicians’ ability to adjust treatment promptly and personalize care. AI-driven tools offer emerging opportunities for predictive modeling and decision support, while telemedicine expands access and convenience. Together, these innovations may support more patient-centered, equitable, and data-informed care.

## 1 Introduction

The rapid evolution of technology, particularly artificial intelligence (AI), is reshaping medical practice across disciplines. In allergy care, digital tools offer opportunities to enhance patient education, refine symptom monitoring, and support more precise diagnostic and therapeutic strategies (1). These innovations may also contribute to greater equity by reducing travel requirements, enabling remote access to specialists, and supporting asynchronous care models, particularly for patients in rural or underserved regions (2, 3).

**Figure 1:**
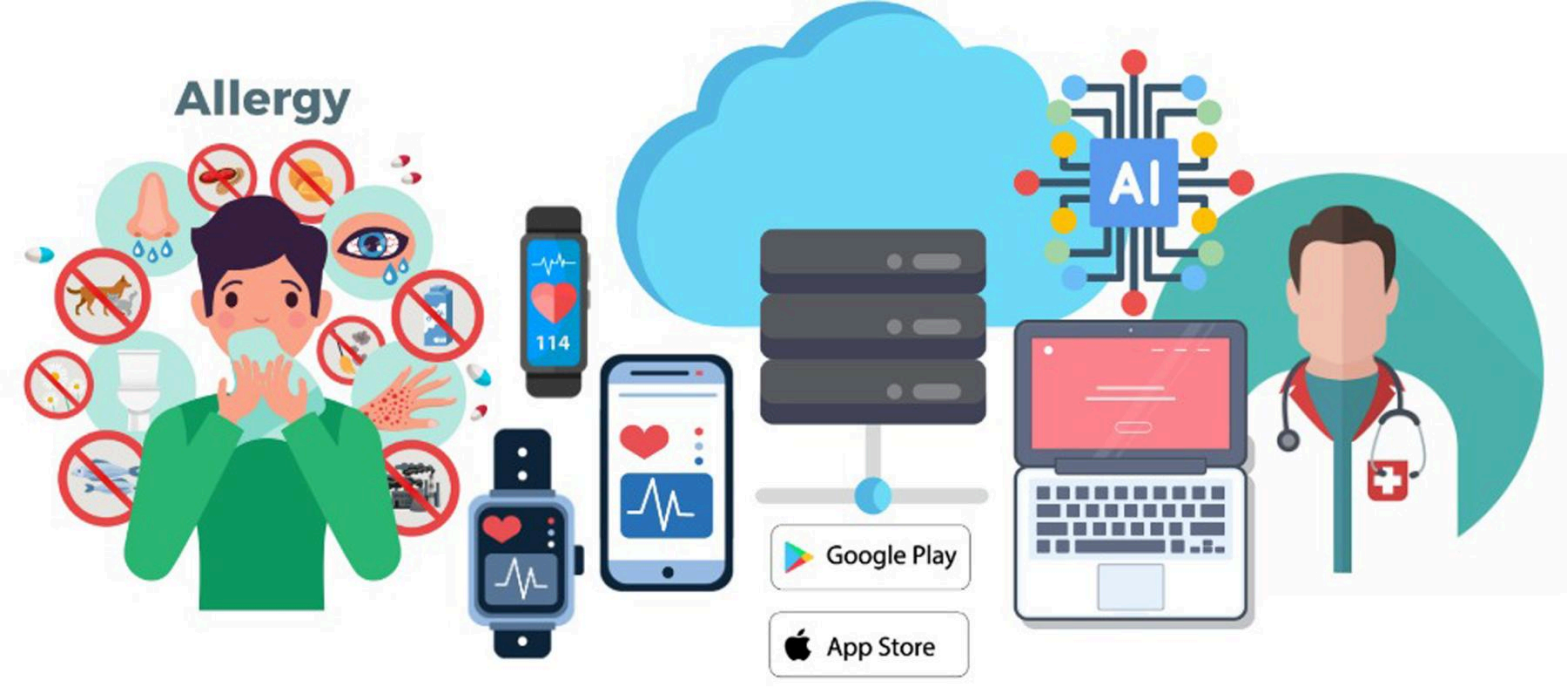
The digital health ecosystem in Allergic Disease.

Digital health encompasses telemedicine, mobile and wearable technologies, remote monitoring systems, and AI-driven decision support, forming an integrated ecosystem linking patients, clinicians, and data streams (Figure 1) (4). As allergic diseases continue to rise globally (5, 6), such approaches are increasingly relevant for improving disease management, optimizing treatment pathways, and strengthening patient engagement.

This mini-review summarizes current developments in digital allergy care, including telemedicine platforms, mobile and wearable self-management tools, and emergent AI-based applications, and discusses the challenges, evidence gaps, and future directions for their integration into clinical practice.

This mini-review is based on a targeted narrative literature search conducted in PubMed, Scopus, and Web of Science, complemented by manual screening of reference lists and relevant guidelines (e.g., ARIA, EAACI). Digital tools included in Tables 1, 2 were selected based on clinical relevance and evidence of validation or real-world implementation.

**Table 1:** Patient-oriented mobile health apps for allergy care.

App name	Developer	Platform	Target Allergy	**Core features**	References
AirRater®	University of Tasmania (Australia)	Android, iOS	AR/AA	Symptoms diary; environmental data	Jones (2021) (52); Workman (2024) (53)
AllergyMonitor®	TPS Software Production (Rome, Italy)	Android, iOS & Web	AA/SAR	Symptoms and medication diary; AIT adherence	Tripodi (2020) (54); Dramburg (2021) (39)
AllergyVax®	BFC Clínica de Saúde (Brazil)	Android, iOS & Web	AA/AR	Immunotherapy management; SLIT adherence	Aarestrup (2025) (55)
AllerSearch	Medical Logue Inc. (Tokyo, Japan)	Android, iOS	AR	Allergy diary, QoL	Inomata (2023) (56)
APOLLO	Munich University and LMU (Germany)	Android, iOS	Pollen-related AR/AA	Patient self-management	Landesberger (2023) (57)
Asthma Health App (AHA)	Mount Sinai School of Medicine & Apple (USA)	iOS (ResearchKit platform)	AA	Patient self-management	Chan (2018) (58)
AsthmaMD	AsthmaMD, Inc. (USA)	Android, iOS	AA	Inhaler adherence	Murphi (2021) (59)
ASTHMAXcel/ASTHMAXcel PRO	Montefiore Applications, LLC	Android, iOS	AA	Patient self-management	Wedel (2024) (60)
Atopic App	Iqutis Labs, Ltd.	iOS	AD	Patient self-management	Zvulunov (2025) (19)
InspirerKids	MEDIDA	Android, iOS	AA, AR	Medication adherence and disease control	Mata (2025) (43)
F3-App	Rhode Island Hospital (USA)	Android, iOS (tablet), computer	FA	Educational software program	Jandasek (2024) (21) NCT05111938
Husteblume (spin-off of Pollen App)	Techniker Krankenkasse, (Hamburg, Germany)	Android, iOS	AR	Patient self-management, pollen forecasts	Glattaker (2020) (44)
juli Health	juli Health (UK/USA)	Android, iOS	AA	Patient self-management	Kandola (2024) (61)
Kiss myAsthma	University of Sydney, Woolcock Institute of Medical Research, University of Melboune (Australia)	Android, iOS	AA	Patient self-management	Davis (2018) (62)
MASK-air® (previously Allergy Diary)	ARIA Initiative/MACVIA (INSERM, France)	Android, iOS	AA/AR	Allergy diary, control assessment, patient self-management, QoL, air quality	Caimmi (2017) (63); Bousquet (2018) (64)
Pollen App	Pollendiary (Austria)	Android/iOS	Pollen-related AR	Allergy diary, patient self-monitoring, pollen forecasts	Bastl (2018) (65)
Propeller Health	Propeller Health (EUA)	Android, iOS + Sensor	AA		Merchant (2016) (10)
SkinTracker	University of California, San Francisco (USA)	iOS	AD	Skin disease monitoring, dermatology clinical research	Marquez-Grap (2025) (66)

This table summarizes selected patient-facing apps for allergic rhinitis (AR), allergic asthma (AA), and related conditions. Columns include app name, developer, platform, target condition, core features, and validation status. Validation refers to published observational studies, randomized controlled trials (RCTs), or feasibility testing. AR, allergic rhinitis; AA, allergic asthma; AIT, allergen immunotherapy; COPD, chronic obstructive pulmonary disease; FA, Food Allergy; QoL, quality of life; SAR, seasonal allergic rhinitis.

**Table 2:** Clinician-oriented platforms and decision-support tools.

Tool name	Developer	Platform	Target Condition	Core features	References
@IT2020-CDSS		algorithm	AR	Shared decision-making (clinics)	Arasi (2021) (31)
AllergyMonitor® (website)	TPS Software Production (Rome, Italy)	Website	AA/SAR	Clinician point-of-care drug allergy diagnosis	Tripodi (2020) (54)
Drug Allergy App	Univ. of Manchester/Birmingham City Univ. (UK)	iOS, Android	DA (β-lactam)	Point-of-care CDSS integrated into an electronic medical record	Elkhalifa et al. (2021) (32)
eAMS	University of Toronto, Canada; St. Michael's Hospital, Canada	iOS	AA	CDSS integrated into an electronic medical record	Gupta (2019) (30)
MASK-air® e-CDSS	ARIA Initiative/MACVIA (INSERM, France)	Web/EHR integrated	AA/AR	Shared decision-making (clinics)	Courbis (2018) (67)
SkinTracker	University of California, San Francisco (USA)	web platform	AD	Researcher-facing portal for clinical trials and teledermatology	Marquez-Grap (2025) (66)

This table lists digital platforms designed for healthcare providers in allergy care. Columns include platform name, developer, type, target condition, core features, and published clinical studies or implementation trials references. AA, allergic asthma; AD,  atopic dermatitis; AR, allergic rhinitis; CDSS, clinical decision support system; DA, drug allergy; HER, electronic health records.

## 2 Telemedicine platforms & virtual consultations

Telemedicine, encompassing synchronous video consultations, telephone visits, e-consults, and remote monitoring, has become an established component of allergy practice (7). It improves timely access to specialists, facilitates follow-up for chronic conditions, and supports ongoing patient education (8). Importantly, telemedicine can advance equity by improving access for rural and underserved populations, provided that broadband connectivity and device availability are addressed (2, 3).

When technical requirements are met, clinical outcomes for asthma and allergic rhinitis are comparable to in-person management, with studies in pediatric and adult populations demonstrating stable disease control and, in some cases, improved treatment adherence (9, 10). Patient-reported satisfaction is consistently high, particularly regarding convenience and communication (8, 11).

Telemedicine also enables structured follow-up in selected subspecialty contexts. Hybrid approaches for food allergy immunotherapy management, in which dose escalation occurs in the clinic and subsequent dose adjustments are conducted remotely, have shown favorable safety in retrospective evaluations (12). Telemedicine can support longitudinal asthma management, including assessment of control, inhaler technique coaching, trigger review, and medication reconciliation using the patient's own devices on camera (13). In dermatology, photo-based evaluations and real-time video review allow prompt adjustment of therapy for atopic dermatitis and chronic urticaria, minimizing delays during flares (14).

Virtual care provides additional value by offering clinicians a direct view of environmental exposures and medication practices in the home. Video-based assessment can verify inhaler technique, confirm medication supplies, and support personalized environmental control counseling (8). Increasingly, these strategies are incorporated into hybrid care pathways, in which virtual encounters are used for interval monitoring and education, while in-person visits are reserved for examinations and procedures requiring physical presence, such as spirometry, auscultation, or skin testing (15).

## 3 Mobile applications, self-management tools, and wearables

Mobile health (mHealth) solutions, including smartphone applications (apps), connected inhaler sensors, home spirometers, fractional exhaled nitric oxide (FeNO) devices, and consumer wearables, allow patients to record symptoms, medication use, and environmental exposures in real time (16). These tools convert intermittent clinic encounters into continuous, patient-generated data streams that support a more proactive and personalized management approach. Importantly, multilingual and culturally adapted apps are essential to ensure equitable adoption, as language and regional restrictions can otherwise perpetuate disparities (3). Across systematic reviews, interactive features such as feedback, reminders, and tailored guidance are consistently associated with improved adherence and better symptom control in asthma and allergic rhinitis (9, 17).

MASK-air, one of the most widely deployed allergy-focused platforms, has generated large real-world datasets across multiple countries and languages. Its combined symptoms and medication scores and digital control indices have contributed to refined phenotyping and informed updates to international guidance, illustrating how standardized patient-generated data can enhance both individual care and population-level insights (18).

Digital tools are also emerging in atopic dermatitis and food allergy, though the supporting evidence remains more limited. Early studies indicate that structured educational content and app-based severity assessments may improve caregiver engagement in pediatric atopic dermatitis (19). In food allergy, available apps show variable quality and often lack clinical validation, although recent user-centered prototypes demonstrate potential for future integration into care pathways (20–22).

Connected device programs complement these approaches. Electronic inhaler monitors paired with smartphone applications and clinician feedback have shown sustained improvements in controller adherence and reductions in rescue medication use in pragmatic and randomized trials (23). Home spirometry and portable FeNO devices further extend remote monitoring capabilities, allowing airway status to be assessed between visits and informing more precise therapeutic adjustments (16, 24, 25). However, home-based measurements such as spirometry and FeNO are subject to user variability, improper technique, and device-related inconsistencies, which may affect data accuracy and require appropriate training and periodic validation.

Educational portals (e.g., EUFOREA's Asthma Portal) with vetted content further support structured self-management. Structured self-management refers to guided patient engagement in monitoring and behavioral adjustments within clinician-defined care plans, rather than independent treatment modification. Vetted content refers to evidence-based educational materials derived from validated sources and international guidelines, including inhaler technique guidance, trigger avoidance strategies, and medication adherence support. In aggregate, mHealth enables a blended model in which patients generate high-resolution data and clinicians translate these data into personalized, timely therapeutic adjustments.

Table 1 summarizes representative patient-facing mobile health applications used in allergic rhinitis, asthma, atopic dermatitis, and food allergy, including core functionalities and available validation data.

## AI and decision support systems

Artificial intelligence (AI) and machine learning (ML) are increasingly applied in allergy care to support prediction, phenotyping, and clinical decision-making (26). Prediction in this context includes forecasting disease exacerbations, estimating treatment response, and identifying high-risk patient phenotypes. These methods operate on heterogeneous datasets integrating clinical variables, biomarkers, environmental information, and patient-generated digital data (27). Beyond clinical analytics, AI-enabled tools can streamline documentation and administrative processes, allowing clinicians to focus more effectively on communication and shared decision-making. (28).

Validated applications are beginninghave begun to emerge in specific domains. In food allergy, ML models trained on well-characterized clinical variables have achieved high discriminative accuracy for predicting oral food challenge outcomes, suggesting a path to reduce unnecessary challenges and improve safety (26, 29). In asthma, guideline-aligned electronic clinical decision support systems (CDSS), such as the Electronic Asthma Management System (eAMS) (30), have improved the standardization of severity assessment and treatment recommendations in primary care settings. These examples illustrate how structured algorithms can reinforce evidence-based practice and reduce unwarranted variability in management.

Additional opportunities are under active investigation. Natural language processing (NLP) systems and large language models (LLMs) are being evaluated for patient education, triage, and documentation support, though clinical oversight remains essential to ensure accuracy and safety (28, 29). Electronic health record–embedded tools may eventually provide automated prompts for drug–allergy interactions, phenotype-based immunotherapy selection, or early identification of patients requiring specialist referral (31, 32). Patient-facing conversational agents may augment inhaler coaching and symptom triage, extending clinician guidance between scheduled encounters (26, 30).

Table 2 summarizes representative clinician-oriented platforms, CDSS tools, and emerging AI applications currently implemented or piloted in allergy practice. Collectively, these technologies highlight a gradual transition from exploratory models to integrated systems designed to enhance decision-making while preserving human oversight.

## 5 Challenges and limitations

Despite the growing adoption of digital tools in allergy care, several barriers limit their reliability, scalability, and clinical impact.

### 5.1 Technical barriers

Technical issues remain a major obstacle to routine implementation. Inconsistent device performance, connectivity problems, and platform incompatibilities can disrupt virtual consultations or interfere with data transmission from remote monitoring tools. Protecting patient-generated health information (symptom diaries, lung function metrics, images, and videos) requires strict compliance with regulatory frameworks such as the Health Insurance Portability and Accountability Act (HIPAA, United States) and the General Data Protection Regulation (GDPR, European Union). This includes robust anonymization or pseudonymization, explicit patient consent, and clearly defined limits on third-party data transfers. Interoperability is another persistent challenge, as many electronic health record (EHR) systems and telehealth platforms lack standardized data structures or application programming interfaces (APIs) that enable seamless exchange of information with remote monitoring and AI tools, resulting in unstructured workflows that place additional burdens on clinicians and limit integration into clinical workflows (33, 34). Adoption of standards such as Health Level Seven (HL7) and its modern specification Fast Healthcare Interoperability Resources (FHIR), along with the World Health Organization (WHO) SMART Guidelines, can enable structured, secure data exchange and harmonized digital health implementations (35, 36). Tool validation is uneven; while, as an example, some asthma apps have demonstrated efficacy, many others lack empirical validation and, in some cases, violate privacy best practices (37). Discordant pollen indices across apps underline the risks of relying on non-validated digital measures (38). Moreover, data volume does not guarantee accuracy, as the quality of patient-entered data cannot be inferred from quantity alone (39). Routine technical failures may also disrupt care. Finally, clinicians must avoid over-reliance on generative AI, which may produce plausible but incorrect outputs; human verification is essential (28).

### 5.2 Equity and access issues (digital divide, literacy, demographics)

Digital care models rely on access to devices, stable broadband connections, and a baseline level of digital literacy. These requirements may disadvantage older adults, patients with lower socioeconomic status, or individuals living in rural areas, potentially widening existing health disparities (40). Financial barriers also play a role, as mobile data costs, subscription-based applications, and device affordability may limit access, particularly in low- and middle-income countries. Algorithmic fairness remains a concern, as models trained on homogeneous datasets may underperform in minority populations, as seen in food allergy prediction models and asthma phenotyping algorithms (27). A large digital asthma program reported smaller improvements and lower engagement among African American participants, highlighting structural inequities (41). Health and digital literacy barriers persist; many patients question the value of mHealth, express privacy concerns, or struggle with device use. Design must address population-specific needs: pediatric tools should involve caregivers, yet few target younger children (42, 43), while older adults benefit from simplified interfaces. Digital maturity is uneven across allergy domains: asthma and rhinitis are relatively well served, while food allergies and atopic dermatitis have fewer validated options. Sustained engagement remains a challenge. Users frequently discontinue apps when symptoms improve, and continuous daily data entry may be burdensome (44). Adaptive, self-learning tools that minimize manual entry and forecast symptom risk may improve retention (38).

### 5.3 Clinical practice barriers (workflow integration, reimbursement)

Integration of digital tools into routine clinical workflows remains challenging for both clinicians and patients. Remote patient-generated data are rarely integrated into standardized processes, leading to inefficiencies and unclear responsibilities. Financial models continue to evolve, although pandemic-era policies expanded telehealth coverage, reimbursement for remote monitoring, asynchronous care, and AI-augmented services remain uncertain (40). Virtual care cannot replace all in-person procedures, such as skin testing, auscultation, and spirometry, limiting its standalone role. Technical failures and variable digital literacy can degrade visit quality and erode confidence (11).

### 5.4 Ethical considerations (privacy, algorithmic bias, dehumanization)

Digital systems raise important questions about privacy, consent, and data stewardship. Telemedicine and AI-enabled tools require secure data storage, transparent data use policies, and clear communication with patients about what information is collected and how it is shared (45). The increasing involvement of for-profit technology companies raises concerns about ownership and secondary use of patient-level data. These entities have an ethical obligation to ensure transparency, equitable benefit sharing, and alignment with public health priorities. Robust governance frameworks are therefore essential to regulate digital health technologies, including standards for validation, auditability, and post-market surveillance. In addition, algorithmic bias remains a critical concern, as models trained on non-representative datasets may perpetuate existing health inequities unless actively monitored using fairness metrics and diverse populations (46).

Beyond data governance, the integration of AI into clinical care raises important questions regarding autonomy, trust, and the nature of the clinician-patient relationship. Transparency in AI-generated recommendations is essential to maintain patient trust; however, such tools should support, rather than replace, clinical decision-making. Reliance on AI without appropriate clinician oversight may pose risks to patient safety, lead to inappropriate treatment decisions, and create uncertainty regarding accountability. Accordingly, human oversight remains essential in all clinical applications.- Furthermore, the limited interpretability of many deep learning models continued to challenge transparency, as current explainability methods only partially address this limitation. The potential dehumanization of care is an additional concern, as overly mechanized or screen-mediated interactions can impair communication and diminish trust, particularly when AI-generated recommendations conflict with clinical judgement. Patient preferences may vary: while some individuals value increased autonomy and reduced clinician interaction, others express discomfort with diminished personal contact. The considerations underscore the importance of flexible, hybrid care models that preserve human engagement while integrating digital innovation (46).

### 5.5 Evidence gaps (limited RCTs, heterogeneity)

Despite the rapid proliferation of digital health tools, rigorous evidence supporting their effectiveness and safety in allergy care remains incomplete. Many studies of digital allergy tools are small, single-center, or observational; high-quality randomized controlled trials are scarce (47). Outcomes often rely on patient-reported measures with limited long-term follow-up. Heterogeneity across platforms, features, and outcome definitions complicates comparison and meta-analysis, limiting firm conclusions about effectiveness and generalizability. In pediatrics, reviews have not conclusively established the equivalence of telemedicine to in-person care across all indications (42). Moreover, numerous health apps lack expert involvement or alignment with medical evidence, reinforcing concerns about safety and real-world impact. The lack of rigorous validation and standardized evaluation frameworks remains a central barrier to widespread, confident adoption.

## 6 Discussion

Digital health has rapidly expanded in allergy care, offering tools such as telemedicine, mobile apps, wearables, and AI-driven decision support systems. Evidence suggests that these innovations can improve patient engagement, monitoring, and treatment adherence, particularly in asthma and allergic rhinitis, while supporting more proactive disease management. Telehealth has become an established modality in allergy practice, facilitating access and continuity of care. However, important challenges remain. Technical barriers, including data privacy, interoperability, and inconsistent validation, continue to limit scalability. Equity concerns, such as the digital divide and algorithmic bias, may further affect inclusivity and access. In addition, evidence gaps persist, particularly regarding long-term outcomes, cost-effectiveness, and applicability across diverse allergy domains. Despite these limitations, digital health tools hold considerable potential to enhance personalized care and complement traditional clinician–patient interactions when implemented within well-defined clinical and regulatory frameworks.

### 6.1 Clinical implications

For allergists, digital health should be viewed as an adjunct rather than a replacement for in-person care (48). Clinicians must participate actively in the development of Request for Comments (RFC)-like standards for data structures, APIs, and communication protocols for mHealth and allergy-specific software (49). Clinicians can begin by integrating validated tools into practices, such as MASK-air for rhinitis or remote peak-flow monitoring for asthma, while maintaining rigorous oversight of data quality and privacy. Telemedicine protocols should be standardized for interventions like oral immunotherapy dose escalation. Training in digital literacy will be essential to preserve patient trust and communication. Clinicians should also leverage patient-generated data to personalize treatment plans, while remaining vigilant about algorithmic limitations and ensuring human verification of AI outputs (50).

### 6.2 Research priorities

Urgent research needs include large-scale randomized controlled trials to establish the effectiveness and cost-effectiveness of digital interventions across allergy domains beyond asthma. Studies should focus on hybrid care models, standardized outcome metrics (including digital biomarkers), and long-term adherence patterns. Another research priority is addressing the interoperability problem. Comparative effectiveness research is needed to evaluate different platforms and identify best practices. Additionally, investigations into algorithmic fairness, usability in diverse populations, and participatory design approaches will help ensure equitable adoption. Real-world implementation studies should assess integration into clinical workflows and reimbursement models.

### 6.3 Implementation roadmap

Practical steps for adoption include:

- Validation and Selection – Use standardized frameworks to evaluate apps and AI tools for safety, privacy, and clinical relevance.
- Workflow Integration – Embed remote monitoring data into EHRs and establish clear protocols for telehealth visits.
- Training and Education – Equip clinicians with digital literacy skills and train patients on app use and data security.
- Policy and Reimbursement – Advocate for sustainable payment models and regulatory clarity for telehealth and digital therapeutics.
- Equity Measures – Address the digital divide through device subsidies, broadband access, and culturally tailored solutions.

These recommendations are derived from synthesis of current literature, international guidelines, and emerging consensus. Their effective implementation requires multidisciplinary collaboration involving patients, clinicians, policymakers, regulators, data scientists, and technology developers.

### 6.4 Future vision

The next decade will see deeper integration of digital health into allergy care. Hybrid models combining virtual and in-person visits will become standard, supported by AI-driven decision support embedded in EHRs. Emerging concepts such as digital twins (virtual patient models built from real-time data) may enable predictive alerts for exacerbations. In parallel, multi-sensor platforms integrating spirometry, FeNO, activity tracking, and environmental data may further enhance personalized management. Additional technologies, including virtual reality for patient education and conversational agents for symptom triage, are also expected to expand the digital ecosystem. Advances such as federated learning (51) and multi-center collaborations may strengthen the robustness and generalizability of AI models, while guidelines from initiatives such as ARIA and EAACI will essential to standardize best practices (4).

Despite these advances, the integration of digital health into allergy care remains contingent on addressing key challenges, including validation, governance, interoperability, and equitable access. While digital tools offer substantial potential to enhance care delivery, their adoption must be guided by robust evidence, appropriate regulatory oversight, and continued clinician involvement. Ultimately, digital health is likely to support a more patient-centered and data-informed approach, complementing traditional care while preserving the importance of human interaction.
